# Provirus Mutations of Human T-Lymphotropic Virus 1 and 2 (HTLV-1 and HTLV-2) in HIV-1-Coinfected Individuals

**DOI:** 10.1128/mSphere.00923-20

**Published:** 2020-09-30

**Authors:** Karoline Rodrigues Campos, Adele Caterino-de-Araujo

**Affiliations:** a Laboratório de Pesquisa em HTLV, Centro de Imunologia, Instituto Adolfo Lutz, Coordenadoria de Controle de Doenças, Secretaria de Estado da Saúde, São Paulo, Brazil; University of Michigan Medical School

**Keywords:** human T-lymphotropic virus 1, HTLV-1, human T-lymphotropic virus 2, HTLV-2, HIV, coinfection, provirus mutation, sequencing, diagnostic impairment

## Abstract

Provirus mutations of human T-lymphotropic virus 1 (HTLV-1), mostly the lack of the 5′ long terminal repeat (LTR) genomic region, have been described and associated with severe adult T cell leukemia/lymphoma (ATLL), non-sense point mutations with low proviral load, and Western blotting indeterminate results. Until now, no information concerning provirus mutations of HTLV-2 and its consequences, as well as those of HTLV-1/2 in HIV-coinfected individuals, had been described. Therefore, we searched for these mutations in provirus samples of 44 HIV/HTLV-1- and 25 HIV/HTLV-2-coinfected individuals. Using protocols well established for amplification and sequencing of segments of the LTR, *env*, and *tax* regions, we searched for defective type 1 particles that retain LTRs and lack internal sequences and type 2 particles that lack the 5′LTR region. In addition, using as references the prototypes ATK (HTLV-1) and Mo (HTLV-2), we searched for point mutations in the LTR and synonyms and nonsynonymous mutations and non-sense mutations in *env* and *tax* regions. Defective HTLV-1 and HTLV-2 provirus type 1 or 2 was detected in 31.8% of HIV/HTLV-1- and 32.0% of HIV/HTLV-2-coinfected individuals. Synonymous and nonsynonymous mutations were identified mostly in HTLV-2 and associated with lower levels of specific antibodies. No non-sense mutations that resulted in premature termination of Env and Tax proteins were detected. On the contrary, mutation in the stop codon of Tax2a produced a long protein characteristic of the HTLV-2c subtype. The clinical significance of these mutations in coinfected individuals remains to be defined, but they confirmed the lower sensitivity of serological and molecular diagnostic tests in HIV/HTLV-1/2 coinfections.

**IMPORTANCE** HTLV-1 and HTLV-2 are endemic to Brazil, and they have different effects in HIV/AIDS disease progression. HIV/HTLV-1 has been described as accelerating the progression to AIDS and death, while HIV/HTLV-2 slows the progression to AIDS. Provirus mutations of HTLV-1 were implicated in severe leukemia development and in problems in the diagnosis of HTLV-1; in contrast, provirus mutations of HTLV-2 had not been confirmed and associated with problems in HTLV-2 diagnosis or disease outcome. Nevertheless, data obtained here allowed us to recognize and understand the false-negative results in serologic and molecular tests applied for HTLV-1 and HTLV-2 diagnosis. Defective proviruses, as well as synonymous and nonsynonymous mutations, were associated with the diagnosis deficiencies. Additionally, since HIV-1 and HTLV-1 infect the same cells (CD4 positive), the production of HIV-1 pseudotypes with HTLV-1 envelope glycoprotein during HIV/HTLV-1 coinfection cannot be excluded. Defective provirus of HTLV-2 and Tax2c is speculated to influence progression to AIDS.

## Abstract (precis)

HTLV-1 and HTLV-2 are endemic to Brazil, and they have different effects in HIV/AIDS disease progression. HIV/HTLV-1 has been described as accelerating the progression to AIDS and death, while HIV/HTLV-2 slows the progression to AIDS. Provirus mutations of HTLV-1 were implicated in severe leukemia development and in problems in the diagnosis of HTLV-1; in contrast, provirus mutations of HTLV-2 had not been confirmed and associated with problems in HTLV-2 diagnosis or disease outcome. Nevertheless, data obtained here allowed us to recognize and understand the false-negative results in serologic and molecular tests applied for HTLV-1 and HTLV-2 diagnosis. Defective proviruses, as well as synonymous and nonsynonymous mutations, were associated with the diagnosis deficiencies. Additionally, since HIV-1 and HTLV-1 infect the same cells (CD4 positive), the production of HIV-1 pseudotypes with HTLV-1 envelope glycoprotein during HIV/HTLV-1 coinfection cannot be excluded. Defective provirus of HTLV-2 and Tax2c is speculated to influence progression to AIDS.

## INTRODUCTION

Human T-lymphotropic viruses 1 and 2 (HTLV-1 and HTLV-2) were the first human retroviruses discovered by Gallo’s team in the early 1980s (1, 2), and HTLV-1 was the first infectious agent discovered to be carcinogenic and the etiological agent of adult T cell leukemia/lymphoma (ATLL) (3–8). The third human retrovirus, named lymphadenopathy-associated virus (LAV) by Montagneir’s group (9) and HTLV-III by Gallo’s group (10), was later discovered to be the etiological agent of AIDS and then named human immunodeficiency virus (HIV) (11). Unfortunately, because of the impact of the HIV epidemic, no attention was paid to HTLV-1/2 infections despite HTLV-1/2 and HIV often being detected in intravenous drug users (IDU) in the 1980s, and the coinfection had been described to accelerate progression to AIDS in homosexual men (12–14). In addition, HTLV-1 is associated with diseases of high morbidity and mortality, such as ATLL- and HTLV-1-associated myelopathy/tropical spastic paraparesis (HAM/TSP) (8).

Like other retroviruses, the HTLV-1 provirus genome contains the *gag*, *pol*, and *env* structural genes flanked by 5′ and 3′ long terminal repeat (LTR) sequences. In the 3′ portion of the genome is a *pX* region that encodes the Tax, Rex, p21, p12, p13, and p30 proteins as well as the antisense gene encoding the HTLV-1 basic leucine zipper factor (HBZ). Both Tax and HBZ are implicated in the development of ATLL, with Tax initiating cellular transformation and HBZ maintaining virus-induced cellular proliferation (15). The Tax protein is also the primary viral antigen targeted by the host's cytotoxic T-lymphocyte response. Increased HTLV-1 Tax expression induces the expression of various cellular genes, such as those encoding interleukin-2 and interleukin-15, which directly contribute to lymphocyte activation and the immunopathogenesis of HAM/TSP, a chronic progressive neurological disease (16–18).

Provirus sequences of HTLV-1 are relatively stable and less prone to mutations than HIV-1 (19). Two types of defective HTLV-1 provirus were described in the 1990s: type 1, which retains both LTRs and lacks internal sequences, and type 2, which lacks the 5′LTR region (20, 21). Interestingly, infected cells with HTLV-1-defective proviruses proliferate more than those with intact ones (22) and were detected at high frequencies in ATL cells and in aggressive forms of ATL (20, 21, 23). These defective sequences were also identified in asymptomatic carriers and HAM/TSP patients, albeit at low frequencies (22, 24).

In addition, HTLV-1 provirus mutations were associated with low proviral load, HTLV-1/2 seronegative screening results, and HTLV-1 Western blotting (WB) indeterminate results (25–27). For instance, defective HTLV-1 provirus in a group of HAM/TSP seronegative patients was described in Chile and Argentina (25, 27). Furthermore, when the proviral loads of HTLV-1 WB-positive blood donors and pregnant women and of HTLV-indeterminate carriers were quantified, a lower proviral load was detected in WB-indeterminate carriers along with an association with proviruses mutations, including non-sense mutations in Pol and/or Tax, Env, p12, and p30 (26). The premature termination of such proteins and the production of abortive strains were considered by the authors to interfere with the host recognition of HTLV-1 antigens and, consequently, the WB indeterminate results (26).

At present, no data concerning provirus mutation and defective particles were described for HTLV-2 or HTLV-1/2 provirus mutations in HIV/HTLV-1 and HIV/HTLV-2 coinfection. Given that we carry out the surveillance of HTLV-1 and HTLV-2 subtypes in HIV/HTLV-1- and HIV/HTLV-2-coinfected individuals in São Paulo, Brazil, we decided to conduct the present study to add information concerning this subject by searching for mutations in the LTR, *env*, and *tax* segments employed in HTLV subtyping.

## RESULTS

After the first screening by NCBI genotyping and REGA genotyping, all HTLV-1 sequences were classified as belonging to the Cosmopolitan subtype a of transcontinental subgroup A and sequences of HTLV-2 as HTLV-2a subtype, variant -2c. These classifications were confirmed by phylogenetic tree constructions (see Fig. S1 and S2 in the supplemental material). The characteristics of patients, the GenBank accession numbers of sequences, and the number of point mutations in each genomic region are disclosed in Table 1.

**TABLE 1:** Number of nucleotide substitutions in LTR, *env*, and *tax* regions of HTLV-1 and HTLV-2 proviruses of HIV-coinfected patients from São Paulo, Brazil^*a*^

ID	Sex	Age (yr)	Result^*b*^	Mutation characteristics										
				LTR			*env*				*tax*			
				GenBank accession no.	Total	Promoter	GenBank accession no.	Total	SY	NSY	GenBank accession no.	Total	SY	NSY
1	M	45	HTLV-2	NA			**KY928504**, *KY928510*	7	6	1	**KT351574**	12	6	6
3	F	49	HTLV-1	KM211972	18	2	KT351486	6	5	1	KT351543	9	6	3
4	M	53	HTLV-1	KM211973	16	0	KT351487	6	5	1	KY928595	20	19	1
5	F	59	HTLV-2	KY928545	22	4	KT351558	11	8	3	KT351579	11	6	5
6	F	58	HTLV-1	KM211974	15	0	KT351488, **KT351478**	6	6	0	**KT351515**	11	9	2
7	M	43	HTLV-1	**KT351465**	17	0	**KT351503**	6	6	0	NA			
9	F	60	HTLV-1	NA			KY928491	7	6	1	KY928587	6	4	2
10	F	46	HTLV-1	KM211975	16	0	KT351489	8	8	0	KT351523	9	7	2
11	M	45	HTLV-1	NA			**KT351482**	8	8	0	NA			
13	M	43	HTLV-1	KM211976	15	1	KT351491, **KT351493**, *KT351506*	8	6	2	KY928591, **KT351526**, *KT351539*	8	6	2
16	M	51	HTLV-1	KM211977	20	2	KT351492	8	8	0	KT351525	10	6	4
17	M	46	HTLV-2	**KY928551**	21	3	**KT351563**	9	8	1	**KT351584**	14	7	7
18	F	55	HTLV-2	**KY928531**	19	3	NA				**KY928598**	12	12	0
20	F	45	HTLV-1	**KY928523**, *KT351468*	19	1	**KT351490**, *KT351507*	5	5	0	**KT351524**, *KT351540*	8	5	3
21	F	53	HTLV-1	**KY928525**	17	0	**KT351501**	6	6	0	NA			
22	M	28	HTLV-1	KM211978	17	2	KT351494, **KY928486**	6	5	1	KT351527, **KT351522**	7	5	2
23	F	48	HTLV-1	**KT351461**	17	0	NA				NA			
24	M	53	HTLV-1	NA			NA				*KT351531*	11	9	2
26	F	52	HTLV-1	NA			KT351496, **KY928494**	6	6	0	**KY928593**	10	7	3
27	M	41	HTLV-1	KM211979	18	2	KT351498, **KT351480**	6	5	1	KT351530, **KT351517**	6	4	2
28	F	68	HTLV-2	*KY928536*	20	6	NA				NA			
29	M	43	HTLV-1	KM211980	20	2	KT351499	6	5	1	KT351532	8	6	2
30	M	43	HTLV-1	KM211981	19	2	KT351500, **KT351497**	6	5	1	KT351533, **KT351529**	7	5	2
31	M	62	HTLV-1	KM211982	16	1	KY928495	6	6	0	KT351534	7	5	2
32	M	48	HTLV-1	KM211983	15	0	KY928496, **KT351483**, *KT351502*	6	6	0	**KT351519**, *KT351536*	9	7	2
33	M	62	HTLV-1	KM211984	17	2	KY928497, KT351484, KY928493	6	5	1	KT351535, **KT351520**, *KY928592*	9	6	3
34	F	57	HTLV-1	**KT351460**	20	2	NA				**KY928578**, KY928590	9	6	3
35	M	55	HTLV-1	KM211985	15	0	KY928498, **KT351477**	6	6	0	KT351537, KT351514	10	8	2
36	M	55	HTLV-1	KY928526	17	0	NA				NA			
37	F	45	HTLV-2	**KY928541**, KY928532	21	6	KT351556, KT351551	8	6	2	KT351569	11	6	5
38	M	61	HTLV-2	KY928550, **KY928540**, *KY928549*	19	5	KT351562, **KY928505**, KT351561	7	6	1	KT351583, **KT351577**, *KT351582*	11	6	5
39	M	51	HTLV-1	**KT351462**, *KT351469*	17	0	**KT351481**, *KT351508*	6	6	0	**KT351518**, *KT351541*	9	7	2
40	M	61	HTLV-2	NA			KY928500, **KY928501**	7	6	1	KT351566, **KT351572**	11	6	5
41	M	59	HTLV-2	**KY928538**	20	5	KT351548, **KT351554**	11	8	3	KY928597, **KT351575**	12	11	1
42	M	46	HTLV-2	*KY928548*	21	5	**KY928503**, *KT351560*	10	8	2	**KT351573**, *KT351581*	12	6	6
43	F	41	HTLV-2	NA			KT351550	9	8	1	KT351568	11	6	5
44	F	43	HTLV-2	KY928535	21	3	NA				NA			
45	M	43	HTLV-2	**KY928539**, *KY928530*	19	3	**KT351555**, *KT351549*	7	6	1	**KT351576**, *KT351567*	11	6	5
46	F	44	HTLV-1	**KT351466**, *KT351459*	23	2	**KT351504**, *KT351479*	6	5	1	**KT351544**, *KT351516*	8	6	2
47	M	48	HTLV-2	KY928537	19	3	KY928502	7	6	1	NA			
50	F	50	HTLV-2	KY928552	21	3	KT351557, **KT351564**	7	6	1	KT351578, **KT351585**	11	6	5
51	F	25	HTLV-1	KT351453	19	0	NA				NA			
52	M	43	HTLV-1	KT351454	20	2	KT351470	8	7	1	KT351509	7	5	2
54	F	48	HTLV-1	KY928513	18	0	KT351471	6	6	0	NA			
55	F	45	HTLV-2	KY928528	25	6	KY928499	11	9	2	KY928596	11	11	0
56	M	53	HTLV-1	KT351455	21	0	KT351472	6	6	0	KT351510	11	9	2
57	F	51	HTLV-2	NA			KT351545	7	6	1	NA			
60	M	18	HTLV-1	NA			KT351473	6	5	1	KT351542	5	3	2
61	F	24	HTLV-1	KT351456	18	2	KT351474	5	4	1	KT351511	7	4	3
64	F	52	HTLV-2	KY928529	24	6	KT351547	7	6	1	KT351565	10	6	4
65	F	43	HTLV-1	KT351457	19	2	KT351475	6	5	1	KT351512	6	4	2
66	F	27	HTLV-1	KT351458	20	2	KT351476	7	6	1	KT351513	7	5	2
68	M	48	HTLV-1	KT351463	18	0	KT351485	5	5	0	KT351521	10	8	2
71	M	59	HTLV-2	KY928547	23	6	KT351559	9	8	1	KT351580	10	6	4
72	F	45	HTLV-1	KT351464	20	2	KT351495	6	5	1	KT351528	8	5	3
78	M	48	HTLV-1	KY928527	20	2	KT351505	8	7	1	KT351538	8	6	2
81	M		HTLV-2	KY928533	23	6	KT351552	9	8	1	KT351570	11	7	4
82	M	61	HTLV-2	KY928534	25	6	KT351553	9	8	1	KT351571	10	6	4
84	F	32	HTLV-1	KY928515	16	0	KY928483	3	3	0	KY928581	6	4	2
86	F	27	HTLV-1	KY928516	16	0	KY928484	4	4	0	KY928582	4	2	2
87	M	49	HTLV-1	KY928517	14	0	KY928485	4	4	0	KY928583	4	2	2
88	F	43	HTLV-1	KY928518	18	1	KY928487	7	6	1	KY928584	7	5	2
89	F	64	HTLV-2	KY928542	22	5	KY928506	8	7	1	KY928599	11	6	5
90	F	53	HTLV-2	KY928543	24	6	KY928507	9	8	1	KY928600	10	6	4
91	F	63	HTLV-1	KY928520	17	1	KY928488	7	6	1	KY928594	17	15	2
92	F	51	HTLV-1	KY928521	17	0	KY928489	3	3	0	NA			
95	F	64	HTLV-2	KY928544	22	5	KY928508	8	7	1	KY928601	11	6	5
100	M		HTLV-1	KY928522	13	0	NA				KY928588	8	6	2
101	M	49	HTLV-2	KY928546	21	5	KY928509	7	6	1	KY928602	11	6	5

a M, male; F, female; ID, identification code of patients according to a previous study (28); SY, synonymous nucleotide substitution; NSY, nonsynonymous nucleotide substitution; NA, not amplified. Nucleotide substitutions are provided as aligned with the ATK prototype (HTLV-1-infected cell line; GenBank accession number J02029) and Mo prototype (HTLV-2-infected cell line; GenBank accession number M10060). GenBank accession numbers of new sequences are provided. Different font styles in GenBank accession numbers mean first blood collection (roman font), second blood collection (boldface), and third blood collection (italics).

b Result obtained after serological and molecular diagnosis of HTLV-1 and HTLV-2.

10.1128/mSphere.00923-20.1 FIG S1 HTLV-1 phylogenetic trees based on the 619-bp nucleotide sequence of the LTR region (A), 619-bp nucleotide sequences of the *env* codifying region (B), and 972-bp nucleotide sequences of the *tax* codifying region (C), using the maximum likelihood approach with 1,000 bootstrap replications, repeated 10 times. The clades supported by bootstrap values of at least 70% are marked with a dot. Branches belonging to Transcontinental subgroup A of the Cosmopolitan subtype a (HTLV-1aA) are in dark blue; branches belonging to Japanese subgroup B of the Cosmopolitan subtype a (HTLV-1aB) are in yellow; branches of subgroup C of the Cosmopolitan subtype a (HTLV-1aC and GH78) are in light blue; branches of subgroup D of the Cosmopolitan subtype a (HTLV-1aD, BO, and OD) are in orange; the branch of subtype b (HTLV-1b, EL, and outgroup in panel C) is in green; the branch of subtype c (HTLV-1c, Mel5, and outgroup in panel A) is in purple; the branch of subtype d (HTLV-1d and pyg19) is in pink; and the branch of STLV, in black, was as an outgroup in panel B. Sequences of the present study are in red. Download FIG S1, DOCX file, 1.6 MB. Copyright © 2020 Campos and Caterino-de-Araujo. 2020 Campos and Caterino-de-Araujo This content is distributed under the terms of the Creative Commons Attribution 4.0 International license.

10.1128/mSphere.00923-20.2 FIG S2 HTLV-2 phylogenetic trees based on the 467-bp nucleotide sequence of the LTR region (A), 1,041-bp nucleotide sequences of the *env* codifying region (B), and 894-bp nucleotide sequences of the tax codifying region (C), using the maximum likelihood approach with 1,000 bootstrap replications, repeated 10 times. The clades supported by bootstrap values of at least 70% are marked with a dot. Branches belonging to subtype a (HTLV-2a, Mo) are in yellow; branches belonging to subtype b (HTLV-2b, NRA) are in green; branches of subtype c (HTLV-1c, BR) are in blue; and the branch of subtype d (HTLV-2d, Efe2), in orange, was used as an outgroup. Sequences obtained in the present study are in red. Download FIG S2, DOCX file, 2.9 MB. Copyright © 2020 Campos and Caterino-de-Araujo. 2020 Campos and Caterino-de-Araujo This content is distributed under the terms of the Creative Commons Attribution 4.0 International license.

Even after several attempts, only part of the DNA samples from 44 HIV/HTLV-1-coinfected individuals was amplified and sequenced for the three LTR, *env*, and *tax* regions (30 [68.2%]), and defective particles of type 1 were detected in 20.4% (considering the lack of *env* and/or *tax* regions) and type 2 in 11.4% (considering the lack of the 5′LTR region) (Table 1). The same deficiencies of sequencing were observed for HTLV-2, in which, among 25 HIV/HTLV-2 samples, 17 (68.0%) were sequenced for the three regions. Defective type 1 particles were detected in 16.0% of samples (considering the lack of *env* and/or *tax* region) and defective provirus type 2 in 16.0% of samples (Table 1). It is important to note that the sequences obtained from the same patient during 2 years of follow-up have been named in different fonts, and although they have received different GenBank access numbers, they are identical, so they were analyzed for mutations only once.

**FIG 1:**
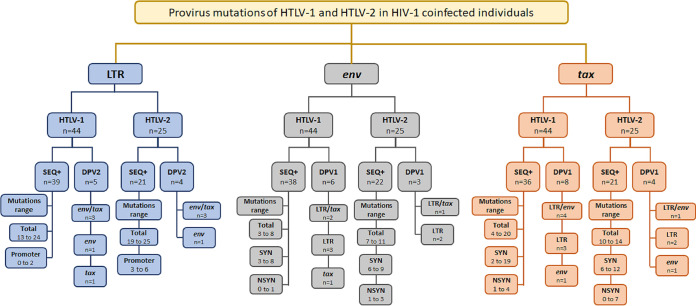
Flowchart of results of genetic sequencing of the LTR, *env*, and *tax* regions and provirus mutations of HTLV-1 and HTLV-2 in HIV-1-coinfected individuals. SEQ+, number of sequences obtained for each region; DPV1, defective provirus type 1; DPV2, defective provirus type 2; Total, range of overall mutations in each region; Promoter, location of the point mutation in the promoter region of the LTR; S, synonymous mutations in the *env* and *tax* regions; NS, nonsynonymous mutations in the *env* and *tax* regions.

Figure 1 summarizes the number of sequences obtained here (SEQ+), the range of provirus point mutations in each region, the location of the point mutation in the promoter region of the LTR, and synonymous (S) or nonsynonymous (NS) point mutations in the *env* and *tax* regions, as well as the number of defective proviruses classified as type 1 (DPV1) and type 2 (DPV2).

**FIG 2:**
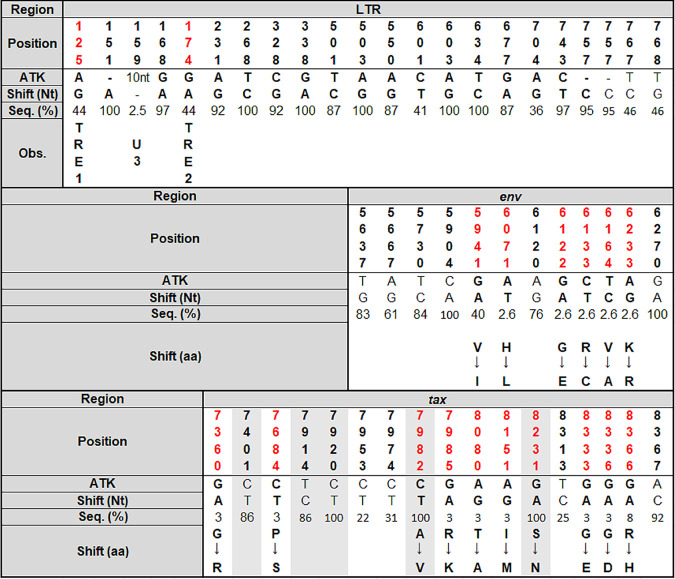
Nucleotide substitutions, insertions, and deletion in the LTR region and nucleotide and amino acid substitutions in *env* and *tax* regions of HTLV-1 sequences. Nt, nucleotide; aa, amino acid; Seq., sequence; Obs., observation. The nucleotide position in the LTR (*n* = 39), *env* (*n* = 38), and *tax* (*n* = 36) regions of HTLV-1 sequences obtained from HIV/HTLV-1-coinfected individuals from São Paulo, Brazil, using the ATK prototype as a reference (GenBank accession number J02029), are disclosed. The promoter regions and regions with nonsynonymous substitutions are in red. The nucleotides and amino acid changes in the *tax* region characteristic of the TaxA Brazilian genotype are highlighted.

Nucleotide substitutions, insertions, and deletions in the LTR of HTLV-1 (including the Tax-responsive elements [TRE1 and TRE2] and U3) and the synonymous and nonsynonymous mutations in *env* and *tax* regions are presented in Fig. 2, highlighting the nucleotide and amino acid changes in the *tax* region characteristic of the TaxA Brazilian genotype.

**FIG 3:**
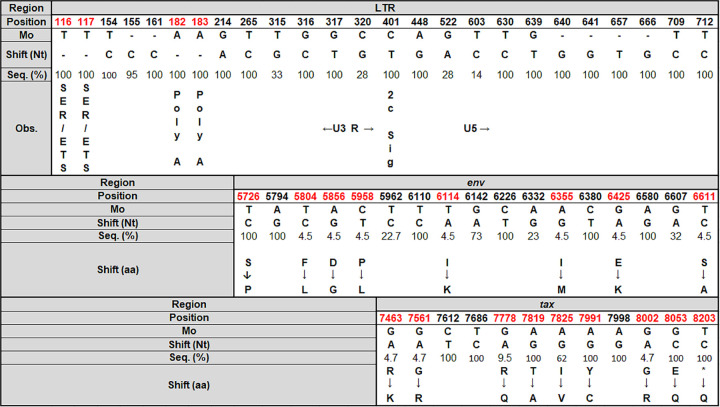
Nucleotide substitutions, insertions, and deletion in the LTR region and nucleotide and amino acid substitutions in *env* and *tax* regions of HTLV-2 sequences. Nt, nucleotide; aa, amino acid; Seq., sequence; Obs., observation. The nucleotide position in the LTR (*n* = 21), *env* (*n* = 22), and *tax* (*n* = 21) regions of HTLV-2 sequences obtained from HIV/HTLV-2-coinfected individuals from São Paulo, Brazil, using the Mo prototype as a reference (accession number M10060), are disclosed. Regions of SER (serum response element), ETS (E-twenty-six transcription factor family), poly(A) (domain responsible for activating mRNA polyadenylation), U3 (3′ unique region in 5′LTR), R (repeat region in 5′LTR), U5 (5′ unique region in 5′LTR), and nonsynonymous substitutions, including the change in the stop codon of Tax2a, giving the molecular signature of HTLV-2c, are highlighted in red.

Figure 3 presents the nucleotide substitutions, insertions, and deletions in the LTR of HTLV-2, including the serum response element (SRE), E-twenty-six transcription factor family (ETS), poly(A), and the signature of -2c in the LTR region, along with synonymous and nonsynonymous mutations in *env* and *tax* regions, emphasizing that the signature of Tax2c (long Tax) was detected in 100.0% of sequences.

**FIG 4:**
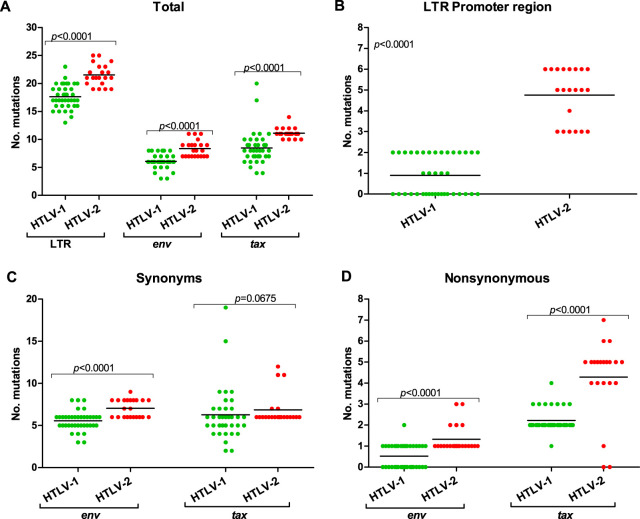
Comparative analyses of HTLV-1 and HTLV-2 considering the overall number of mutations, point mutations in the LTR promoter region, and synonymous and nonsynonymous mutations in the *env* and *tax* regions using the Mann-Whitney U test.

Comparative analyses of the number of mutations detected in the LTR, *env*, and *tax* regions of HTLV-1 and HTLV-2 are presented in Fig. 4. They confirm more mutations of HTLV-2 than HTLV-1 in HIV/HTLV-coinfected individuals (*P < *0.0001 in all cases, except synonymous mutations in the *tax* region) (Fig. 4C). Of note, more mutations were observed in the LTR region (more than 15 in HTLV-1 and more than 20 in HTLV-2) (Fig. 4A).

The impact of such mutations and the presence of defective proviruses were evaluated in relation to anti-HTLV-1/2 antibody production using samples previously classified as HTLV-1 and HTLV-2 and independently confirmed by serologic (Western blotting and/or line immunoassay) or molecular assays (qPCR [*pol*] and nested PCR-restriction fragment length polymorphism [RFLP] [*tax*]) (28). Differences in the levels of antibodies (optical density to cutoff [OD/CO] values), depending on the applied HTLV-1/2 screening immunoassays, were detected.

**FIG 5:**
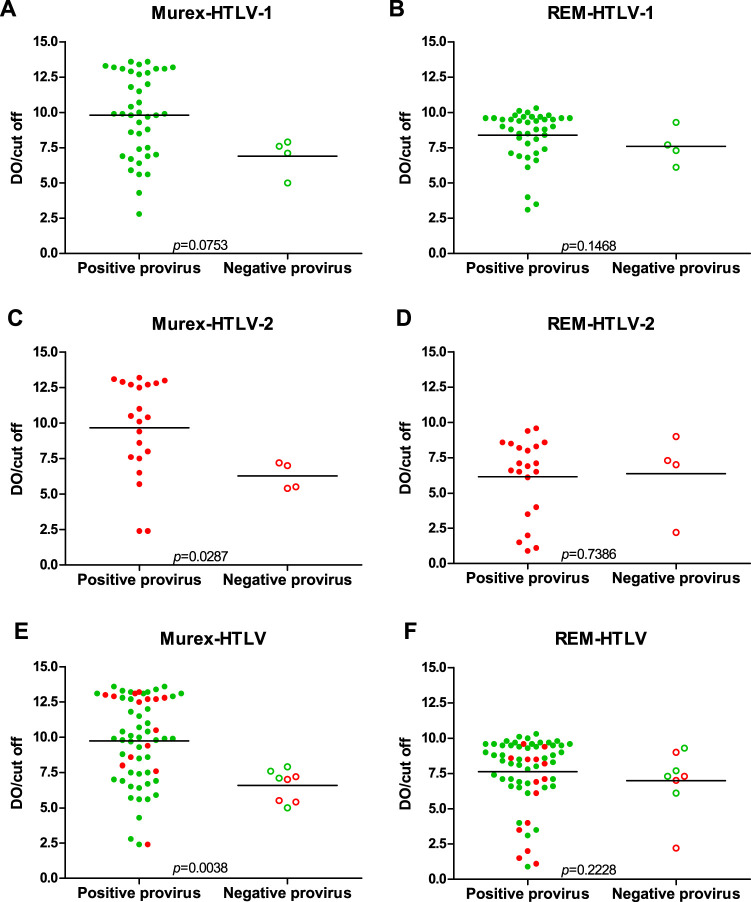
Comparative analyses of HTLV-1/2 antibody levels (OD/CO) using two enzyme immunoassays in relation to provirus detection by molecular assays (quantitative PCR [*pol*] and nested PCR-RFLP [*tax*]). Statistical analyses used the Mann-Whitney U test.

Figure 5 presents the comparative analysis of the OD/CO values obtained from two enzyme immunoassays (Murex EIA HTLV-I/II [Diasorin, United Kingdom] and Gold enzyme-linked immunosorbent assay [ELISA] HTLV-I/II [REM Indústria e Comércio LTDA, São Paulo, Brazil]) and the results of molecular confirmatory assays. Four of the 44 HTLV-1 samples and 4 of the 25 HTLV-2-negative samples in the confirmatory molecular assays had lower average levels of antibodies than the positive ones when the Murex EIA was used for screening, with statistical significance for HTLV-2 and HTLV-1 plus HTLV-2 samples (Fig. 5C and E). In contrast, no difference in antibody levels and negative and positive confirmatory molecular assays was detected when the REM Gold ELISA was employed for screening (Fig. 5B, D, and F).

**FIG 6:**
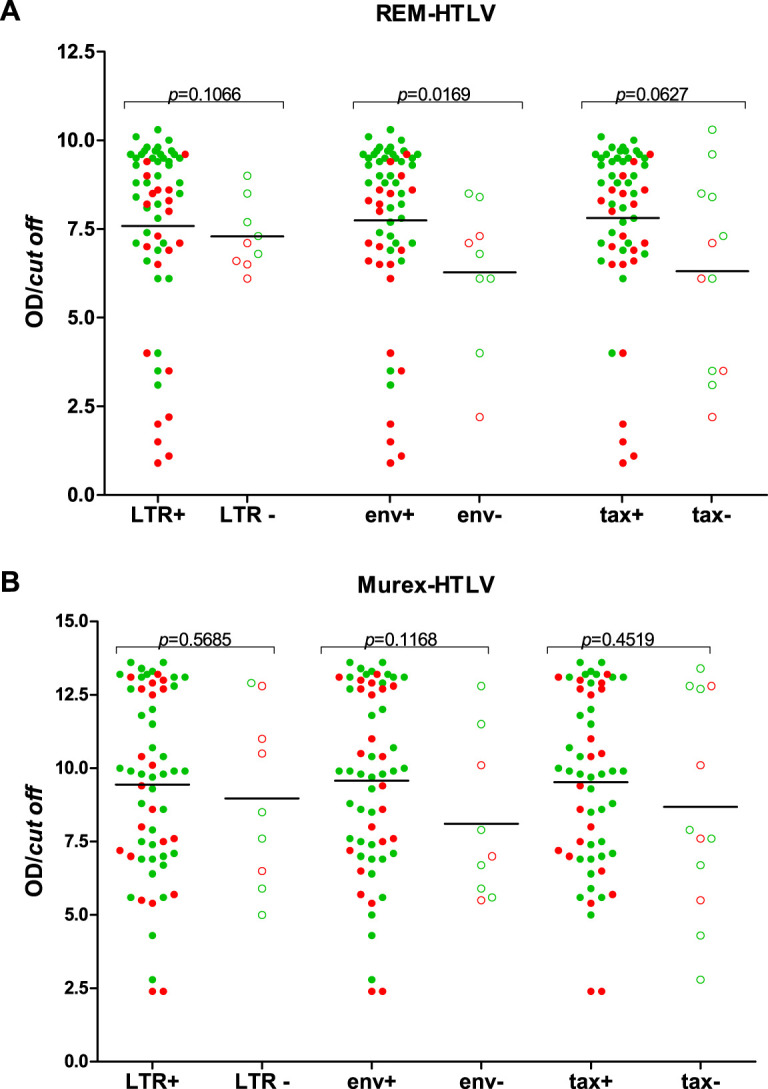
Comparative analyses of HTLV-1/2 antibody levels (OD/CO) using two enzyme immunoassays in relation to the positivity of LTR, *env*, and *tax* sequences, using the Mann-Whitney U test.

On the other hand, for analyses of average OD/CO values of both serological screening assays and the results of the presence or absence of defective provirus in LTR, *env*, and *tax* sequences (positive or negative), although overall comparisons disclosed lower levels of antibodies in blood samples that had negative sequence results, statistically significant difference was detected only for the *env* region and when REM Gold ELISA was used for screening (Fig. 6A).

**FIG 7:**
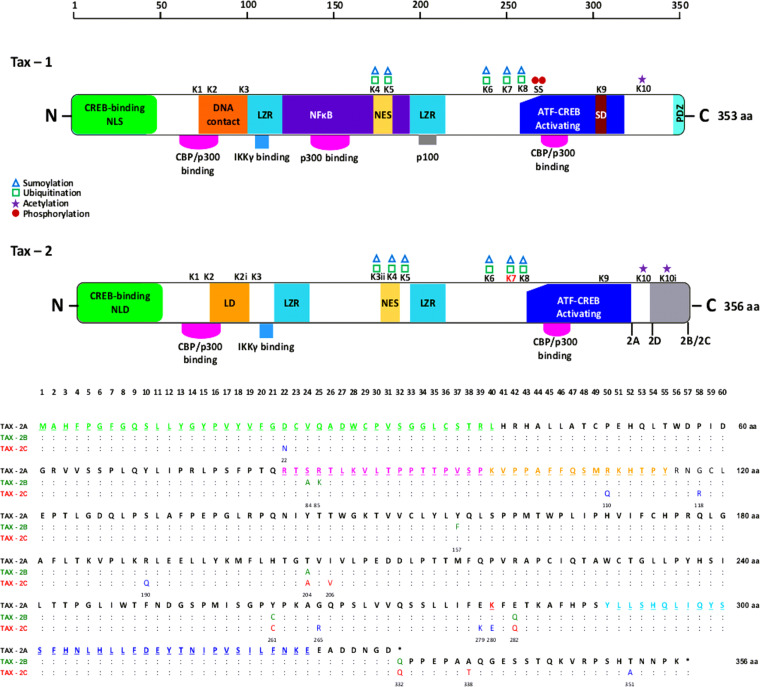
Structural and functional domains of the Tax-1 and Tax-2 proteins and amino acid sequence alignment of the Tax2 genotypes (Tax-2A, Tax-2B, and Tax-2C). K, position of lysine; S, position of serine; CBP, Ca^2+^ binding protein; NLS, nuclear localization signal; LZR, leucine zipper-like regions; NES, nuclear export sequence; SD, secretion domain; NLD, nuclear localization determinant; LD, localization domain. The PDZ-binding motif, CREB-binding region, and activating transcription factor (ATF) are shown. Amino acid changes in dark green belong to genotype Tax-2B. Amino acid changes belonging to genotype Tax-2C are in red (present in the majority of sequences) and dark blue (present in the minority of sequences).

Finally, Fig. 7 presents the structural and functional domains of Tax-1 and Tax-2 and the amino acid sequence alignment of the Tax-2 genotypes (Tax-2A, Tax-2B, and Tax-2C), emphasizing the amino acid substitution mostly in Tax-2-specific domains that may affect cellular localization or posttranslational modification and the lack of the stop codon that characterizes the Brazilian HTLV-2c variant of HTLV-2.

## DISCUSSION

The primary objective of the present study was to search for provirus mutations and/or defective proviruses of HTLV-1 and HTLV-2 circulating in HIV/HTLV-coinfected individuals to know if they influence HTLV-1/2 diagnosis and to speculate whether these mutations could interfere with virological and patient outcomes. For these attempts, we analyzed sequences employed in HTLV-1 and HTLV-2 subtype surveillance in São Paulo, Brazil.

The results of HTLV-1 and HTLV-2 subtyping showed Cosmopolitan subtype a of the Transcontinental subgroup A of HTLV-1 (HTLV-1aA) and the variant, c, of the HTLV-2a subtype (HTLV-2c) circulating in São Paulo, confirming the HTLV subtypes more frequently detected in Brazil in individuals of African origin, Amerindians, and IDUs (29–33).

Concerning nucleotide substitutions in LTR regions, the results obtained showed nucleotide substitutions in TRE1 and TRE2, as previously described (34, 35). The implication of such substitution was not clarified, although it was tentatively implicated in HTLV-1 proviral load regulation (34). Curiously, in HTLV-2 sequences, more mutations in the LTR region were detected here, and the proviral load of HTLV-2 was low compared with that of HTLV-1 described in the literature (36). In fact, in HTLV-2-infected individuals, less positivity in serologic and molecular confirmatory assays has been described and associated with low proviral load (28).

Other mutations were detected in the LTR genomic region, such as the C401T mutation, which confirms the molecular signature of the HTLV-2c subtype and mutations in the SER, ETS, poly(A), and U3RU5 regions, as reported by Barreto and coworkers in viral isolates from Salvador, Bahia, in the northeast region of Brazil (37). Interestingly, some of these transcription factors can activate the cell cycle, inducing apoptosis and cell growth and participating in the malignancy of tumor cells and metastasis. Furthermore, poly(A) has an important role in mRNA regulation, stability, and translation (38–40). Thus, mutations in these transcription factor motifs may interfere in cellular and viral component production and could be related, in some way, to the protective role of HTLV-2 in HIV-coinfected individuals, especially in cases infected by HTLV-2c.

Regarding synonymous and nonsynonymous mutations in the *env* regions of HTLV-1 and HTLV-2, again, greater numbers of mutations were detected in HTLV-2. The amino acid change S183P in 100.0% of the HTLV-2c *env* sequences was also previously reported in Brazilian strains (29, 41, 42) and confirmed the Brazilian signature of strains. The *env* mutations plus the LTR mutations detected in HTLV-2 allowed us to suppose that mutations in LTR promoter regions plus mutations in the *env* region could be associated with minor antigen production and, consequently, antibodies. In fact, in the present study, more mutations in LTR, *env*, and *tax* regions of HTLV-2 than of HTLV-1 were detected, mostly in the LTR promoter region.

Additionally, the presence of mutations in *env* of HTLV-1, similar to the absence of this segment in some strains, does not eliminate the possible presence of pseudotypes of HIV and HTLV-1, since both retroviruses infect the same cell type (CD4^+^ cells). Moreover, the unique lack of the *env* region detected in some samples in the present study allowed us to suppose pseudotype production. Tang and collaborators described pseudotyping of HIV-1 with HTLV-1 envelope glycoprotein during HIV-1/HTLV-1 coinfection, facilitating direct HIV-1 infection of female genital epithelial cells and implicating it in the sexual transmission of HIV in nonpermissive genital cells (43). This phenomenon may represent a risk factor for enhanced sexual transmission of HIV-1 in regions where HIV/HTLV-1 coinfection is common, as in Brazil. The presence of such hybrid particles could not be excluded in this study and could be responsible, in part, for the fast progression to AIDS and death of HIV/HTLV-1-coinfected individuals described elsewhere (44–46). Of note, during the period of the present study, no patient developed ATLL or HAM/TSP or died of AIDS.

In addition, although recombinant forms of HTLV-1 have been described only in some African strains (47), we could not exclude recombinant forms of HTLV-1 as well HTLV-2 in coinfected individuals, as well as pseudotyping of HIV and HTLV-1/2.

An important piece of data that emerged from the present study was the strong association of the low titers of HTLV-1/2 antibodies when the Murex EIA was employed for screening with the negative results, mostly for HTLV-2, using PCR of *pol* and *tax* segments as confirmatory diagnostic assays. Defective type 1 particles, which include the lack of *pol*, *tax*, and/or *env* regions, and point mutations in such regions could account for the results obtained. The Murex EIA contained only recombinant glycoproteins of the envelope (rgp 46-I and rgp 46-II) and the transmembrane glycoprotein GD-21, common to both viruses, and just in this segment there is an amino acid change of S183P in HTLV-2 *env* sequences. We hypothesized that defective provirus type 1 particles or nonsynonymous mutations in the *env* region are responsible for the results obtained. On the other hand, when the same antibody levels were evaluated in relation to the detection of LTR, *env*, and *tax* proviral segments of HTLV-1 and HTLV-2, although minor levels of antibodies were detected in both EIAs in the overall analysis, only the REM Gold ELISA results reached statistical significance (association between the absence of *env* sequences and low levels of antibodies). We do not know which are the synthetic peptides or recombinant proteins present in this REM EIA, because this information is absent from the manufacturer’s instructions, but we hypothesized that when nonsynonymous point mutations and defective proviruses are circulating in such individuals, small amounts of antibodies are produced and, consequently, detected by this enzyme immunoassay. In fact, the lower sensitivity of this screening assay was previously described in HIV/HTLV-coinfected individuals in São Paulo, Brazil, when the comparative performance of Murex EIA and REM Gold ELISA was examined (28), corroborating the present data. On the other hand, no differences in the levels of antibodies were detected using the REM Gold ELISA samples that were positive and negative in molecular confirmatory assays. Again, the antigen composition of both assays could explain the discordant results obtained here.

Of note, false-negative results in molecular assays were detected in the present study, as were false-negative results in serologic screening assays described in HTLV truly infected individuals from South America (25, 27, 28). Thus, although in this study no blood samples that could be amplified and sequenced had a false-negative result in the screening, there is a need to improve the commercially available serological screening tests for HTLV in South America, perhaps using chimeric antigens containing *env* (rgp46), *gag* (p19), and *pX* (Tax) proteins (27).

Regarding Tax protein, Brazilian molecular signatures were detected in both HTLV-1 and HTLV-2. Overall, HTLV-1 isolates disclosed mutations that confirm the molecular signature of the TaxA genotype, characteristic of Brazilian strains described by Kashima and coworkers (48) and corroborated by other groups in HTLV-1-infected (49) and HIV/HTLV-1-coinfected individuals of Brazil (50). Of note, in Brazil, no association of this genotype and HTLV-1-associated diseases was detected (49).

Concerning Tax of HTLV-2, studies conducted by Bertazzoni’s group have demonstrated that Tax-1 and Tax-2b have differences in transformation and expression capacities; they possess two basic structural features that differentiate Tax-1 from Tax-2, i.e., the presence only in Tax-1 of an NF-κB2 domain and a PDZ binding motif, responsible for the binding to p100 and several PDZ-containing proteins. Tax-1 and Tax-2 also have several common domains but divergent cellular localization. Tax-1 predominates in the nucleus, while Tax-2b predominates in the cytoplasm of cells (51). When we analyzed the Tax protein of the Brazilian variant (HTLV-2c) present in individuals from urban areas, we detected Tax-2c in all sequences.

In a preliminary study conducted by Bertazzoni’s team and our group comparing 25 Tax-2c sequences obtained from patients of Brazil with Tax-2b sequences obtained from patients in Italy, we found that they differed for amino acid substitutions in 11 different positions. These substitutions may affect cellular localization or posttranslational modification and confirmed the CREB-binding site in the N-terminal region and the ATF/CREB activating domain in the C-terminal region and an additional localization domain responsible for cytoplasmic cellular localization, similar to that of Tax-2b (52). In the present study, using other Brazilian HTLV-2 sequences and the alignment of Tax-2c with the proteins Tax-2a and Tax-2b, the same amino acid mutations were detected, requiring further phenotypic studies of the properties and cellular localization of Tax-2c.

Another important detail that emerged from this study was the high rate of defective provirus particles (around 30%) detected in HIV/HTLV-1- and HIV/HTLV-2-coinfected individuals, which is similar to the overall rates described in ATL individuals infected with HTLV-1 (20–22). However, the percentages differ depending on the type of defective provirus (type 1 or type 2). While in HTLV-1-infected individuals the majority of cases are defective provirus type 2 instead of type 1 (26.7% versus 2.2% in ATL, 14.5% versus 2.9% in asymptomatic carriers, and 13.1% versus 2.0% in HAM/TSP, respectively) (22), in the present study we detected more defective type 1 particles in HIV/HTLV-1 coinfection (20.4% versus 11.4%) and the same rates of type 1 and type 2 in HIV/HTLV-2-coinfected individuals (16.0%).

Concerning the limitations of the present study, we take into account that we used a conventional DNA sequencing methodology (Sanger sequencing), which generates provirus DNA fragments (not the complete provirus) present only in the major clones. Although we consider that the best way to search for defective proviruses is using a high-throughput sequencing-by-synthesis instrument (Illumina massively parallel sequencing technology) (32) or a recent viral DNA sequencing capture approach, which amplifies the full-length proviruses and provides information about the proviral structure and the viral integration sites (22), we tried to avert this lack of amplifying large fragments of the 5′LTR, *env*, and *tax* regions used in HTLV subtyping. We employed degenerate primers and protocols optimized previously for detecting all Brazilian sequences described in the literature (29, 35, 50, 53, 54); indeed, we increased the DNA input and repeated the reaction several times, and, to ascertain that the 5′LTR and not the 3′LTR of HTLV-1 was amplified, we employed primers pairs that cover the entire LTR region plus a close fragment of the *gag* gene of 66 bp (nucleotides 756 to 821). We believe these strategies may reduce the limitations of the study.

The implication of the mutations and defective proviruses in HIV/HTLV-1- and HIV/HTLV-2-coinfected individuals detected here deserves more study, but we speculate that difficulties in HTLV-1 and HTLV-2 diagnosis as well negative results in molecular confirmatory assays in such individuals are, in part, attributable to these provirus mutations, corroborating results described elsewhere for HTLV-1-infected individuals (26).

In addition, we could not exclude the negative impact of defective particles in HIV/HTLV-1 coinfection, since low CD4^+^ cell counts, high HIV viral load levels, and more X4 HIV strains were detected in such patients. In contrast, in HIV/HTLV-2-coinfected patients, we detected large numbers of CD4^+^ cells, low HIV viral load levels, and large numbers of R5 HIV strains (all data were from laboratory records). These data partially corroborate the studies that pointed to rapid progression to AIDS and death in HIV/HTLV-1-coinfected individuals and slow progression to AIDS in HIV/HTLV-2-coinfected individuals (44–46). Of note, the DNA samples analyzed in the present study came from patients of the same cohort, with approximately 15 to 20 years of HIV and probably HTLV infections, the majority of whom were on antiretroviral therapy (ART). Thus, the apparent conflicting result of CD4^+^ cells in HIV/HTLV-1-coinfected individuals (since HTLV-1 induces T cell proliferation) could be due to the late starting of ART in such patients, when the recovery of CD4^+^ cells is not promising, but these are only speculations and merit other studies.

In conclusion, this is the first time that around 30% of provirus mutations were described in HTLV-2-infected individuals and in HIV/HTLV-1- and HIV/HTLV-2-coinfected individuals and is the first tentative description of HTLV-2 defective provirus classification. Indeed, the presence of point mutations and defective provirus was correlated with negative results in molecular assays and lower antibody production. Finally, the results obtained here could help us in the future to understand why HIV/HTLV-1 and HIV/HTLV-2 progress differently to AIDS and to HTLV-1/2-associated diseases.

## MATERIALS AND METHODS

The samples analyzed consisted of blood samples obtained from patients with HIV/AIDS attending AIDS Reference Centers in São Paulo, Brazil, and we conducted a comparative analysis of serologic and molecular diagnostic assays described elsewhere (28). Of note, two enzyme immunoassays were employed for HTLV-1/2 screening (Murex EIA HTLV-I/II and Gold ELISA HTLV-I/II). Two serologic assays (WB and HTLV BLOT 2.4; MP Biomedicals Asia Pacific Pte. Ltd., Genelabs), a line immunoassay (INNO-LIA HTLV I/II Score; Fujirebio Europe N.V., Belgium), and two molecular in-house assays (qPCR [*pol*] and nested PCR-RFLP [*tax*]) were used as HTLV-1 and HTLV-2 confirmatory and discriminatory assays (28). Samples were considered truly HTLV-1 or HTLV-2 positive when positive in at least one of these serological and/or molecular assays.

DNA samples were extracted from peripheral blood leukocytes. The protocols for amplification and sequencing the LTR, *env*, and *tax* regions were previously described and employed degenerate primers and protocols optimized for detecting all Brazilian sequences obtained from the literature (29, 53–55). The reactions were conducted in triplicate. The primer pairs employed in the study are disclosed in Table S1 in the supplemental material. The HTLV-1 provirus products amplified varied from 621 to 625 bp (LTR/*gag*), 634 to 679 bp (*env*), and 996 to 1,074 bp (*tax*), and those of HTLV-2 provirus varied from 496 to 644 bp (LTR), 936 to 964 bp (*env*), and 1,089 to 1,134 bp (*tax*). Products available for sequence analysis were obtained from 44 HIV/HTLV-1- and 25 HIV/HTLV-2-coinfected individuals. From some patients in clinical and laboratory follow-up for 2 years, DNA samples were obtained at the beginning of study (first collection), 6 to 12 months later (second collection), and 13 to 24 months later (third collection). The sequences obtained from these patients are presented in Table 1.

10.1128/mSphere.00923-20.3 TABLE S1 Primers employed in polymerase chain reaction assays (LTR, *env*, and *tax*). Legend: a, primer nucleotide position is provided as aligned with the ATK prototype (HTLV-1-infected cell line; GenBank accession number J02029) and Mo prototype (HTLV-2-infected cell line; GenBank accession number M10060); b, primers employed in nested PCR and sequencing reactions. Primers were employed to detect HTLV-1 and HTLV-2 provirus DNA in peripheral blood leucocytes of HIV-coinfected patients from the southeast (São Paulo) region of Brazil. Download Table S1, DOCX file, 0.02 MB. Copyright © 2020 Campos and Caterino-de-Araujo. 2020 Campos and Caterino-de-Araujo This content is distributed under the terms of the Creative Commons Attribution 4.0 International license.

Briefly, sequencing was performed using an ABI 3130 Genetic Analyzer (Applied Biosystems, Foster, CA). All of the sequencing chromatograms were assembled and edited with Sequencher 4.7 software. Multiple alignments were performed using the Clustal W multiple-sequence alignment tool from the BioEdit Sequence Alignment Editor, version 7.0.5.3, and software with a reference set available in the GenBank database (https://www.ncbi.nlm.nih.gov/genbank), in which the nucleotide and amino acid substitutions were searched. HTLV-1 and HTLV-2 subtyping was primarily screened by NCBI genotyping (https://www.ncbi.nlm.nih.gov/projects/genotyping/formpage.cgi) and REGA subtyping (http://www.bioafrica.net/rega-genotype/html/subtypinghtlv.html) websites. Neighbor-joining (NJ) and maximum-likelihood (ML) phylogenetic trees were constructed based on appropriate nucleotide substitution models determined by Modeltest v3.7 (GTR+G model for LTR-1, *env*-*2*, and *tax*-*2*, GTR+I+G for *env*-*1* and *tax*-*1*, and TVM+I+G for LTR-2) using PAUP v4b10 software. Bootstrapping was performed using the stepwise addition algorithm for 1,000 replicates. The Mel5 (HTLV-1c) sequence and Efe2 (HTLV-2b) were used as outgroups. The GenBank accession numbers of the prototypes as well the sequences obtained here and employed in the phylogenetic tree constructions are disclosed in Text S1.

10.1128/mSphere.00923-20.4 TEXT S1 GenBank accession numbers. Download Text S1, DOCX file, 0.01 MB. Copyright © 2020 Campos and Caterino-de-Araujo. 2020 Campos and Caterino-de-Araujo This content is distributed under the terms of the Creative Commons Attribution 4.0 International license.

After confirming the HTLV-1 and HTLV-2 subtypes of the Brazilian sequences, they were aligned and compared with the prototypes ATK (HTLV-1a) and Mo (HTLV-2a) using BioEdit version 7.0.5.3 software and searched for nucleotide substitutions and amino acid changes (synonymous and nonsynonymous mutations). In the *tax* regions of HTLV-1, we searched for the *taxA* and *taxB* genotypes (50). In the *tax* region of HTLV-2, the molecular variant of the Brazilian HTLV-2a strains, named HTLV-2c, which encodes a long transactivating protein (Tax) because of the loss of the stop codon in the *tax* gene position 8203, was used; consequently, an additional 25 amino acids at the C-terminal end of the Tax protein (29) were used.

Of interest, these prototypes were employed for subsequent analyses because they have been used to compose the HTLV-1/2 serologic immunoassays employed for screening, confirmation, and discrimination of HTLV-1 from HTLV-2 (as cell line viral lysates and/or as recombinant protein or synthetic peptides).

It is worth noting that the ATK prototype is the complete nucleotide sequence of the HTLV-1 provirus genome integrated into leukemia cell DNA, derived from a Japanese adult T cell leukemia (ATL) patient (56). The Mo prototype is the complete nucleotide sequence of the HTLV-2 provirus genome integrated into leukemia cell DNA derived from an American patient presenting with hairy cell leukemia (2).

The examination of defective proviruses was conducted according to the lack of LTR, *env*, and *tax* segments after several amplifications and sequencing events, including testing different amounts of DNA inputs and primer pairs. The criterion to be considered an HTLV-1 defective type 1 provirus was the lack of the *env* and/or *tax* segment, and that for type 2 was the lack of a 5′LTR region (20–22). The same criterion used for HTLV-1 was employed for HTLV-2, since there is no publication or classification of defective proviruses of HTLV-2 in the literature.

The possible influences of provirus mutations in the local interaction of the Tax protein and other cellular factors in LTR promoter segments, as well the Env and Tax protein products in antibody production, intracellular Tax localization, and cellular factor interaction of HTLV-1 and HTLV-2, were searched as previously described (37, 51, 55).

GraphPad Prism software, version 5.03 (San Diego, CA, USA), was employed for comparisons among groups using the Mann-Whitney U test (two groups). A *P* value of ≤0.05 was considered significant.

The study was approved by the Ethics Committee for Research of IAL, CTC 62H-2015 and 21I/2016, under Ministry of Health protocol numbers CAAE 55837316.0.0000.0059 and 52493316.1.0000.0059. All of the procedures were performed in accordance with the principles established in the Declaration of Helsinki of 1975, as revised in 1983. The study was conducted anonymously.

### Data availability.

GenBank accession numbers for the prototypes and sequences obtained in this study can be found in supplemental Text S1.
