# Supplementary figures and images for: Provirus Mutations of Human T-Lymphotropic Virus 1 and 2 (HTLV-1 and HTLV-2) in HIV-1-Coinfected Individuals
Source: mSphere. 2020 Sep 30;5(5):e00923-20. doi: 10.1128/mSphere.00923-20 (PMC7529439; doi:10.1128/mSphere.00923-20)

**
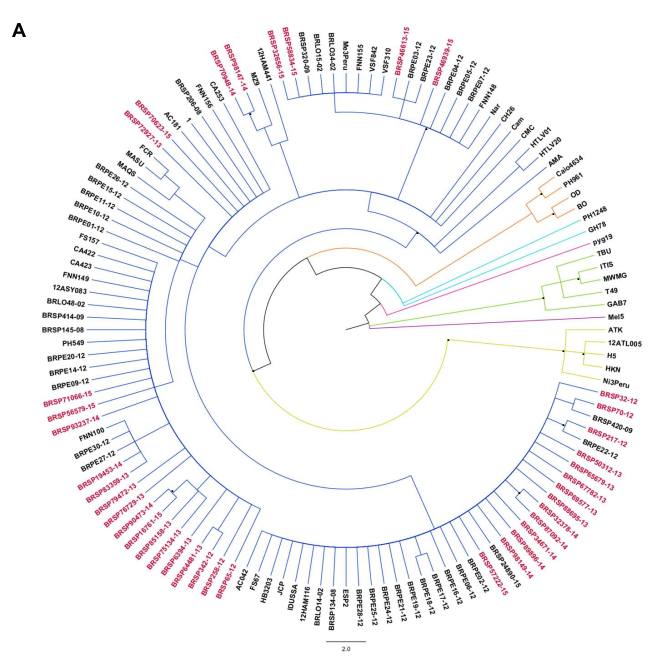
**

**
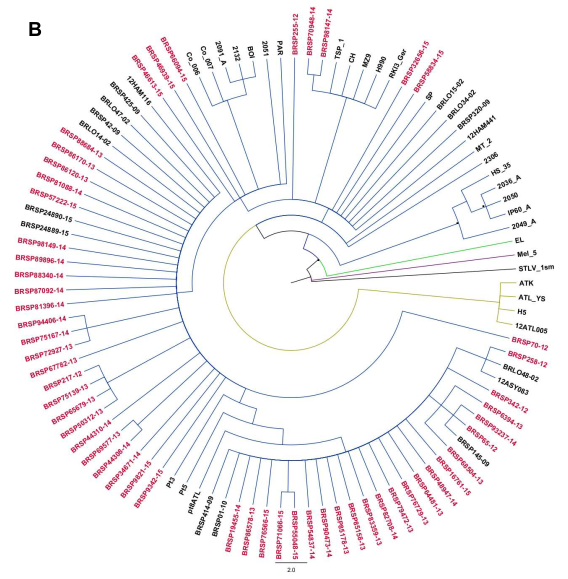
**

**
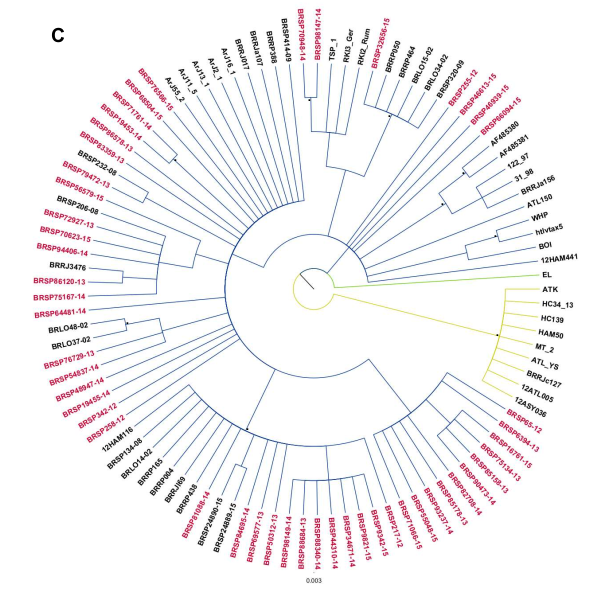
**

Supplement: FIG S1 [file mSphere.00923-20-sf001.docx]

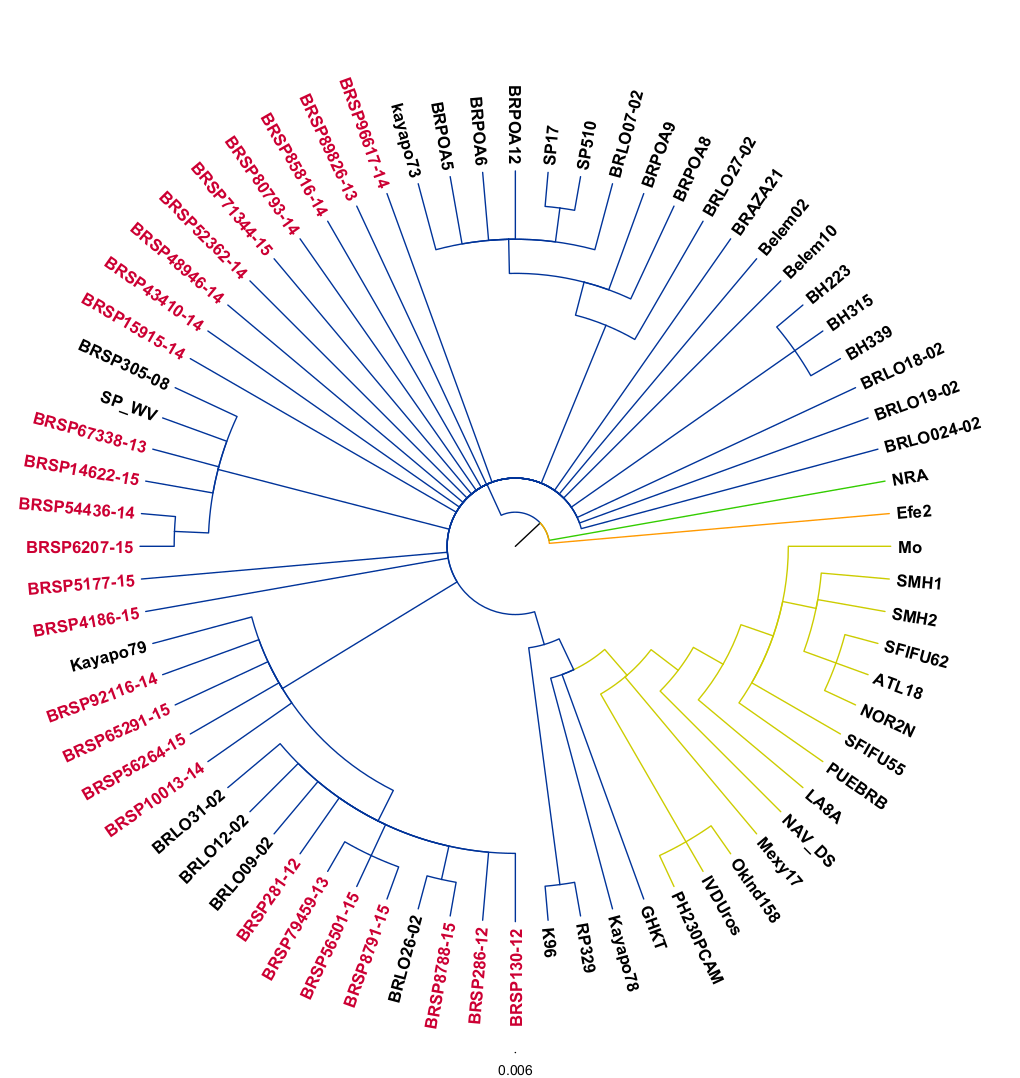


**A**

.

.

.

.

.


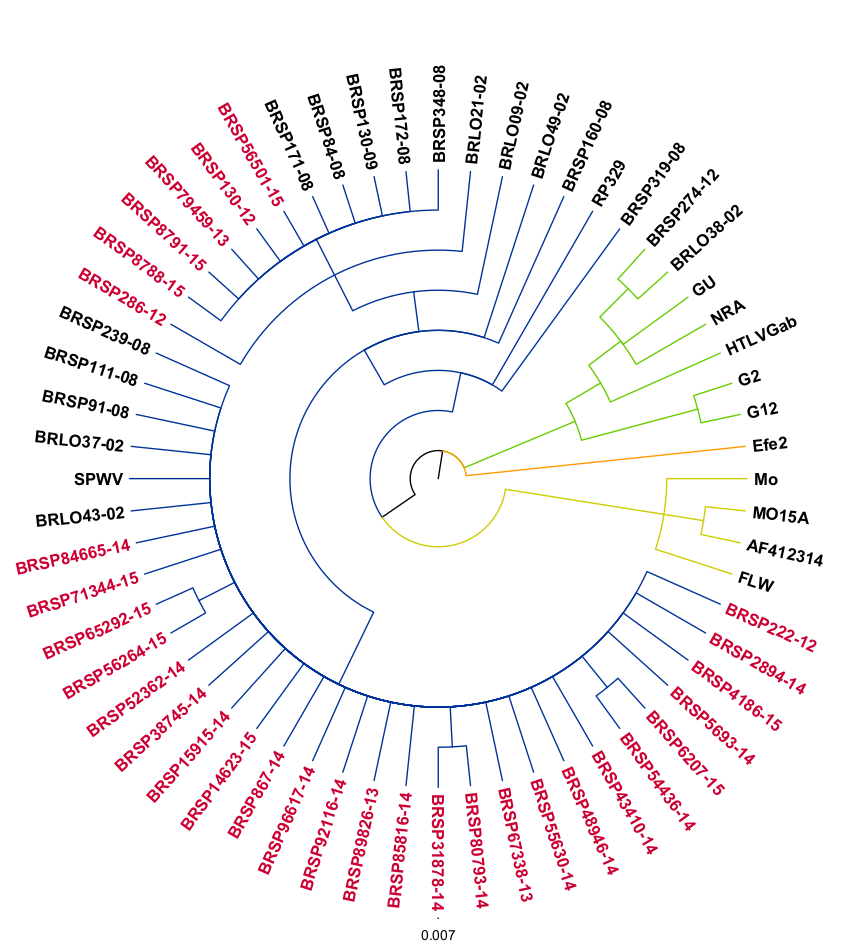


**B**

.

.

.

.

.

.

.


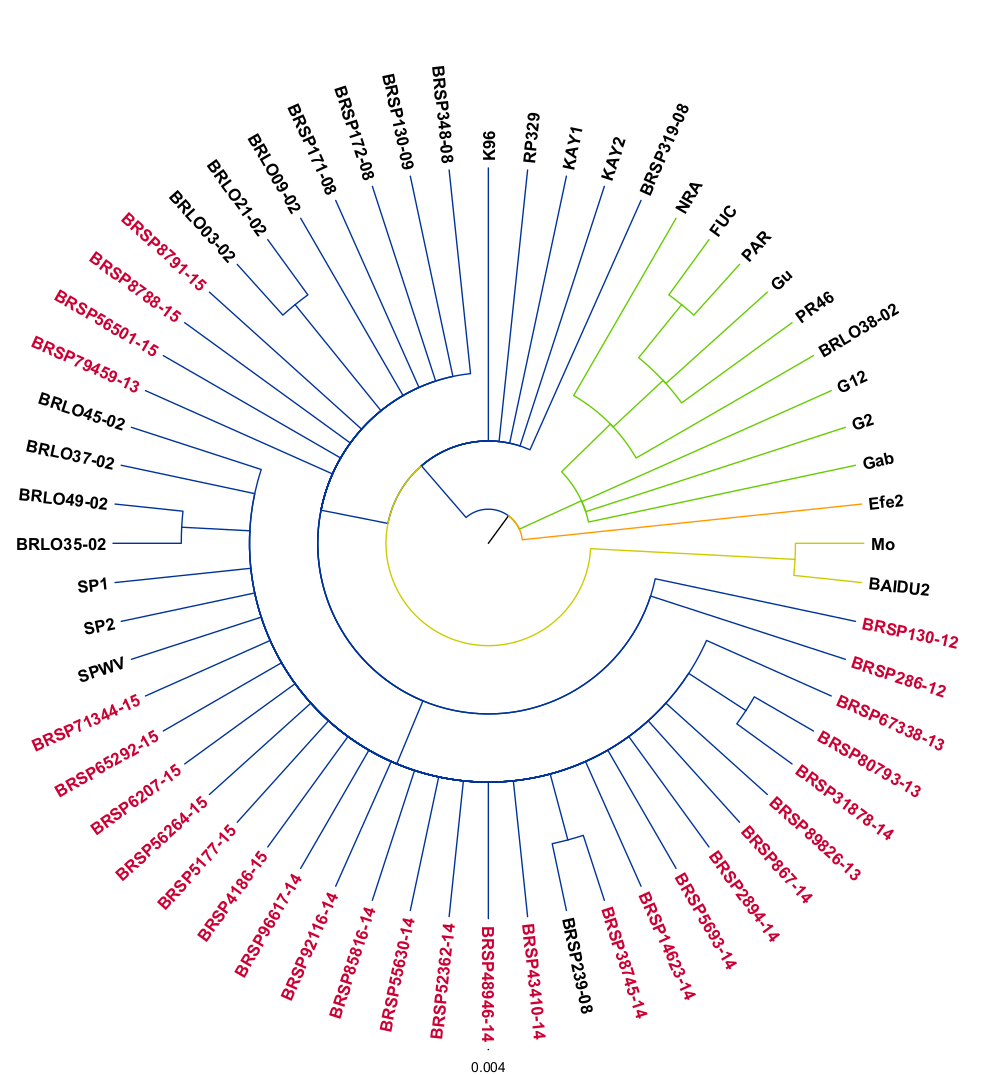


**C**

.

.

.

.

Supplement: FIG S2 [file mSphere.00923-20-sf002.docx]
