# Supplementary material for: Provirus Mutations of Human T-Lymphotropic Virus 1 and 2 (HTLV-1 and HTLV-2) in HIV-1-Coinfected Individuals
Source: mSphere. 2020 Sep 30;5(5):e00923-20. doi: 10.1128/mSphere.00923-20 (PMC7529439; doi:10.1128/mSphere.00923-20)
Supplement: TABLE S1 [file mSphere.00923-20-st001.docx]

| **Target** | | **PCR** | **Primer** | **Sequence** | **Position^a^** |
| --- | --- | --- | --- | --- | --- |
| **HTLV-1** | **LTR** | 1^st^ round | LTR-I.03 Forward | 5'-GGC TTA GAG CCT CCC AGT GA-3' | 57-76 |
|  |  |  | LTR-I.02 Reverse | 5'-CGC GGA ATA GGG CTA GCG CT-3' | 864-845 |
|  |  | nested | LTR-I.03 Forward^b^ | 5'-GGC TTA GAG CCT CCC AGT GA-3' | 57-76 |
|  |  |  | LTR-I.04 Reverse^b^ | 5'-GCC TAG GGA ATA AAG GGG CG-3' | 822-803 |
|  | ***env*** | 1^st^ round | D498 Forward | 5'-ATG GGT AAG TTT CTB GCC-3' | 5202-5219 |
|  |  |  | D500 Reverse | 5'-TTA CAG GGA TGA CTB AGG-3' | 6668-6651 |
|  |  | nested | LUI 7 Forward^b^ | 5'-CCG TCT CCA GYC CMT MCT GG-3' | 5547-5567 |
|  |  |  | LUI 8 Reverse^b^ | 5'-CCT CGT CTR TTY TGG GCW GCA-3' | 6309-6289 |
|  | ***tax*** | 1^st^ round | PXO1 Forward | 5'-TCG AAA CAR CCC TRC AGA TA-3' | 7280-7299 |
|  |  |  | PXO2 Reverse | 5'-TGA GCT TAT GAT TTG TCT TCA-3' | 8470-8490 |
|  |  | nested | PXI1 Forward^b^ | 5'-ATA CAA AGT TAA CCA TGC TT-3' | 7297-7316 |
|  |  |  | PXI3 Reverse^b^ | 5'-AGA CGT CAG AGC CTT AGT CT-3' | 8397-8416 |
|  |  | Sequencing | PXI2 Forward | 5'-GGC CAT GCG CAA ATA CTC CC-3' | 7641-7660 |
|  |  |  | PXI1 Reverse | 5'-GGG TTC CAT GTA TCC ATT TC-3' | 7667-7686 |
|  |  |  | PXI2 Reverse | 5'-GTC CAA ATA AGG CCT GGA GT-3' | 8047-8066 |
|  |  |  | PXI3 Reverse | 5'-AGA CGT CAG AGC CTT AGT CT-3' | 8397-8416 |
| **HTLV-2** | **LTR** | 1^st^ round | VS1 Forward | 5'-CAG GGC GAG TCA TCG ACC CAA AAG-3' | 35-58 |
|  |  |  | VS2 Reverse | 5'-GAA GAC AAT GCT CCT AGG GCG GGC-3' | 746-724 |
|  |  | nested | VS3 Forward^b^ | 5'-ACC GTC TCA CAC AAA CAA TCC C-3' | 64-85 |
|  |  |  | VS4 Reverse^b^ | 5'-GCG GGC CTG CCT ATA GCG ATG-3' | 729-709 |
|  | **env** | 1^st^ round | E5 Forward | 5'-AGC CAA GTG TCC CTT CGA CTA-3' | 5612-5632 |
|  |  |  | E2 Reverse | 5'-CTG CAG AAG CTA GCA GGT CTA-3' | 6670-6650 |
|  |  | nested | E3 Forward^b^ | 5'-TTC TCT AAG TGC GGC TCC TC-3' | 5636-5655 |
|  |  |  | E2 Reverse^b^ | 5'-CTG CAG AAG CTA GCA GGT CTA-3' | 6670-6650 |
|  |  | Sequencing | GP21F1 Forward | 5'-CTG CAA CAA CTC CAT TAT CCT-3' | 6041-6060 |
|  | **tax** | 1^st^ round | PX101 Forward | 5'-GGC AAT CTC CTA AAA TAG TCT-3' | 7155-7175 |
|  |  |  | Px106 Reverse | 5'-GGG CCG TGG TTT CAG TTC CTA-3' | 8306-8326 |
|  |  | nested | PX103 Forward^b^ | 5'-TTA CAA TCC TGT CTC CTC TCA-3' | 8306-8326 |
|  |  |  | Px106 Reverse^b^ | 5'-GGG CCG TGG TTT CAG TTC CTA-3' | 8306-8326 |
|  |  | Sequencing | Px105 Forward | 5'-GCT YTC CCC ACC CAT GAC ATG-3' | 7680-7603 |
|  |  |  | LS1 Forward | 5'-GAA TAC ACC AAC ATC CCT GTC-3' | 8140-8160 |
|  |  |  | Px102 Reverse | 5'-TGT GTG TAG GAA CAT TTT GTA-3' | 7795-7815 |
