# Supplementary material for: Provirus Mutations of Human T-Lymphotropic Virus 1 and 2 (HTLV-1 and HTLV-2) in HIV-1-Coinfected Individuals
Source: mSphere. 2020 Sep 30;5(5):e00923-20. doi: 10.1128/mSphere.00923-20 (PMC7529439; doi:10.1128/mSphere.00923-20)
Supplement: TEXT S1 [file mSphere.00923-20-s0001.docx]

**HTLV-1**

**LTR:** ATK (J02029), pyg19 (L76310), ITIS (Z32527), MWMG (Z31662), T49 (L76305), GAB7 (L76311), GH78 (D23693), OD (U12805), BO (U12804), Ni3.Peru (Y16485), H5 (M37299), HTLV01 (DQ005556), HTLV20 (DQ005564), CH26 (D23690), FNN148 (DQ005548), Nar (AF063820), Me3.Peru (Y16480), FNN155 (DQ005551), Cam (AF063819), IDUSSA (DQ005555), FNN156 (DQ005552), MAQS (X88876), MASU (X88877), JCP (X88875), HKN (X88874), FCR (X88873), CMC (X88872), AMA (X88871), FNN100 (DQ005547), FNN149 (DQ005549), CA253 (EU108722), CA423 (EU108724), CA422 (EU108721), HB3120 (DQ471206), HB3203 (DQ471205), FS157 (GU225732), FS67 (GU225731), VSF842 (EF672337), VSF310 (EF672336), AC042 (EU392160), AC181 (EU392159), Mel5 (L02534), 012BR_HAM116 (KF797912), 012BR_ASY083 (KF797896), 012BR_HAM441 (KF797905), 12BR_ATL005 (KF797887), PH961 (KJ544815), PH1248 (KJ544826), PH549 (KJ544836), TBU (KJ544855), AC181 (EU392159), ES-P2 (GU126483), Caio4634 (JN655856), 1 (KC493378), BRLO14-02 (JF271836), BRLO15-02 (JF271837), BRLO34-02 (JF271839), BRLO48-02 (JF271842), BRSP134-08 (JF271843), BRSP145-08 (JF271844), BRSP206-08 (JF271845), BRSP320-09 (JF271849), BRSP414-09 (JF271850) e BRSP24890-15 (KY928514).

***env:*** ATK (J02029), TSP-1 (M86840), MT-2 (M37747), HS35 (D13784), CH (M69044), 2036-A (AY604874), pt3ATL (U81866), pt5ATL (U81867), pt8ATL (U81868), 2306 (AY604896), MZ9-08 (HM770441), EL (M67514), Co-006 (AF405343), Co-007 (AF405344), 2049-A (AY604875), 2050 (AY604876), 2051 (AY604877), 2091-A (AY604885), 2132 (AY604887), BOI (L36905), ATL-YS (U19949), H5 (M37301), H990 (U81862), IP60-A (AY604934), PAR (AY604935), RK13-Ger (AF042071), SP (M69044), Mel5 (L02534), STLV-1-sm (U94516), 012BR_ASY083 (KF797896), 012BR_HAM441 (KF797905), 12BR_ATL005 (KF797887), BRSP320-09 (HM770437), BRSP414-09 (HM770439), BRSP42-09 (HM770438), BRSP425-09 (HM770440), BRSP01-10 (HM770431) BRLO14-02 (HM770426), BRLO15-02 (HM770427), BRLO34-02 (HM770428), BRLO47-02 (HM770429), BRLO48-02 (HM770430), BRSP24889-15 (KY928481) e BRSP24890-15 (KY928482).

***tax:*** ATK (J02029), TSP-1 (M86840), BRRJ017_95 (DQ323833), BRRJl69_00 (DQ323845), BRRJc127_97 (DQ323840), BRRJ3476 (DQ323834), BRRJa107_96 (DQ323835), BRRP388 (DQ323855), BRRP165 (DQ323875), BRRJa156_971 (DQ323836), BRRP004 (DQ323870), BRRP438 (DQ323862), BRRP050 (DQ323850), BRRP464 (DQ323883), ArJ13-1 (DQ227134), ArJ2-1 (DQ227130), ArJ16-1 (DQ227135), ArJ11-5 (DQ227158), ArJ55-2 (DQ227189), BOI (L36905), WHP (AF259264), htlvtax5 (AF226593), ATL150 (AB036376), RKI3-Ger (AF042071), RKI2-Rum (AF003012), HC34-13 (AB045549), MT-2 (AF292000), HC139 (AB045520), ATL-YS (U19949), HAM50 (AB045481), (AF485380), (AF485381), 31/98 (AF133525), 122/97 (AF133524), EL (M67514), BRLO14-02 (JN887698), BRLO15-02 (JN887699), BRLO34-02 (JN887700), BRLO37-02 (JN887701), BRLO48-02 (JN887702), BRSP134-08 (JN887703), BRSP206-08 (JN887705), BRSP232-08 (JN887706), BRSP320-09 (JN887709), BRSP414-09 (JN887710), 012BR_ASY036 (KF797891), 012BR_HAM441 (KF797905), 12BR_ATL005 (KF797887), 012BR_HAM016_HC (KF797893), BRSP24889-15 (KY928579) e BRSP24890-15 (KY928580).

**HTLV-2**

**LTR:** Mo (M10060), SFIFU62 (U73022), NOR2N (U10258), ATL18 (U10252), SMH2 (Y09148), SMH1 (Y05147), SFIFU55 (U73010), PUEBRB (U10262), LA8A (U10256), NAV.DS (U10257), Oklnd158 (U73015), IVDUros (AF054272), PH230PCAM (Z46838), Mexy17 (L42510), GHKT (L42507), RP329 (AF326583), K96 (AF326584), Kayapo78 (AF139388), SP-WV (AF139382), Kayapo73 (L42509), BRPOA6 (DQ028606), BRPOA12 (DQ028613), BRPOA9 (DQ028608), Belem10 (AF139393), Belem02 (AF139392), BH223 (AY509600), BH339 (AY509602), BRLO7-02 (GU573730), BRLO9-02 (GU573731), BRLO12-02 (GU573732), BRLO18-02 (GU573733), BRLO19-02 (GU573734), BRLO24-02 (GU573738), BRLO26-02 (GU573739), BRLO27-02 (GU573740), BRLO31-02 (GU573743), Kayapo79 (AF1239389), BRPOA5 (DQ028605), BRPOA8 (DQ028607), BRAZA21 (U10253), BH315 (AY509601), NRA (L20734); Efe2 (Y14365).

***env*:** Mo (M10060), BRLO21-02 (HM770399), BRLO22-02 (HM770400), BRLO37-02 (JQ435911), BRLO38-02 (HM770409), BRLO43-02 (HM770411), BRLO49-02 (HM770413), BRSP91-08 (HM770416), BRSP111-08 (HM770417), BRSP160-08 (HM770418), BRSP171-08 (HM770423), BRSP172-08 (HM770423), BRSP239-08 (HM770419), BRSP319-08 (HM770420), BRSP348-08 (HM770425), BRSP84-09 (HM770421), BRSP130-09 (HM770422), G12 (L11456), Gu (X89270), Gab (Y13051), G2 (AF074965), RP329 (AF326583), SP-WV (AF139382), AF412314 (AF412314), MO15A (K02024), FLW (S67545), NRA (L20734), Efe2 (Y14365).

***tax*:** Mo (M10060), K96 (AF326584), G12 (L11456), BAIDU2 (AF401496), SP-WV (AF139382), SP2 (U32872), SP1 (U32873), FUC (U32882), PAR (U32880), KAY1 (U32875), KAY2 (U32874), G2 (AF074965), Gab (Y13051), Gu (X89270), PR-46 (DQ022075), BRLO03-02 (JN887713), BRLO09-02 (JN887714), BRLO21-02 (JN887717), BRLO35-02 (JN887724), BRLO37-02 (JN887725), BRLO38-02 (JN887726), BRLO45-02 (JN887729), BRLO49-02 (JN887730), BRSP171-08 (JN887731), BRSP172-08 (JN887732), BRSP239-08 (JN887733), BRSP319-08 (JN887734), BRSP348-08 (JN887735), BRSP130-09 (JN887736), NRA (L20734), Efe2 (Y14365).
